# Double-seronegative neuromyelitis optica: it is not possible to interrupt treatment after 10 years of stability *

**DOI:** 10.1055/s-0045-1814370

**Published:** 2026-02-04

**Authors:** Vitória Pimentel, Jefferson Becker

**Affiliations:** 1 Pontifícia Universidade Católica do Rio Grande do Sul, Hospital São Lucas, Departamento de Neurologia, Porto Alegre RS, Brazil.; 2 Pontifícia Universidade Católica do Rio Grande do Sul, Faculdade de Medicina, Porto Alegre RS, Brazil.

**Keywords:** Neuromyelitis Optica, Autoantibodies, Immunosuppressive Agents, Relapse, Drug Withdrawal, Aquaporin 4, Biomarkers

## Abstract

Neuromyelitis optica spectrum disorder (NMOSD) is a severe autoimmune demyelinating disease characterized by a high risk of relapse and disability. In cases positive for aquaporin 4-immunoglobulin G (AQP4-IgG), long-term immunosuppression is the established standard. However, the management of double-seronegative NMOSD remains controversial due to limited data. Although some authors propose treatment discontinuation in patients with prolonged remission, there are no reliable predictors of sustained disease inactivity. Given the unpredictability and severity of the relapses, even after a decade of stability, and the lack of robust evidence supporting safe cessation, we argue that maintenance immunotherapy should remain the standard of care in most cases. The risks of disease reactivation outweigh the potential benefits of treatment withdrawal. Until validated biomarkers or clinical tools for individualized risk stratification emerge, the default approach should prioritize sustained disease suppression.

Neuromyelitis optica spectrum disorder (NMOSD) is a relapsing autoimmune disease of the central nervous system that, if inadequately treated, can lead to severe and often irreversible disability. 1 2 Unlike multiple sclerosis (MS), in which older age is associated with declining disease activity and diminished therapeutic response, which has prompted investigations into de-escalation strategies, NMOSD relapses remain unpredictable and clinically-severe across all age groups, including older adults. 3 These attacks frequently result in profound neurological deficits such as blindness or paraplegia, and recovery after relapses tends to be incomplete even when promptly treated with high-dose corticosteroids or plasma exchange. Complete recovery only occurs in approximately 1/3 optic neuritis attacks, leaving approximately 2/3 patients with permanent visual sequelae, while more than 80% of longitudinally-extensive transverse myelitis (LETM) episodes result in long-term motor, sensory, or sphincter dysfunction. 4

For patients positive for aquaporin 4-immunoglobulin G (AQP4-IgG), indefinite immunosuppressive therapy is widely accepted as necessary to prevent relapse and cumulative disability. 2 3 4 However, for patients who are double-seronegative, negative for AQP4-IgG and myelin oligodendrocyte glycoprotein-immunoglobulin G (MOG-IgG), the optimal duration of treatment remains undefined. This seronegative NMOSD population is heterogeneous. 5 While AQP4-IgG-positive patients often exhibit more aggressive disease, with greater female predominance, higher lesion burden, and increased association with other autoimmune disorders, 5 6 7 seronegative patients may present with a broader clinical spectrum. These individuals more frequently experience bilateral optic neuritis, simultaneous optic neuritis and myelitis, and, occasionally, a monophasic disease with better recovery and less long-term disability, particularly in overlapping MOG antibody-associated disease (MOGAD) cases. 6 8 This clinical and prognostic heterogeneity raises questions about the most appropriate therapeutic decision for these patients.

Nevertheless, multicenter studies 9 have shown that seropositive and seronegative patients benefit comparably from immunosuppressive treatment in terms of relapse reduction. Recent trials 10 11 with satralizumab and inebilizumab, though primarily enrolling AQP4-IgG-positive patients, showed significant relapse reduction in the overall NMOSD population. Efficacy was greatest among AQP4-IgG–positive patients; yet, the observation of benefit in seronegative subgroups reinforces that immune modulation addresses common mechanisms across NMOSD variants. Satralizumab reduced THE relapse risk by approximately 55 TO 62%, and inebilizumab achieved a 73% relative reduction compared to placebo, 10 11 12 reinforcing the need for continued immunosuppression in seronegative NMOSD. Unfortunately, most randomized trials and consensus guidelines focus almost exclusively on AQP4-IgG-positive populations, leaving treatment decisions for seronegative cases dependent on extrapolated data. 13 The international diagnostic criteria do include seronegative cases based on clinical and radiological findings, but these patients remain underrepresented in therapeutic research. 14

Thus, accurate diagnosis of double-seronegative NMOSD is crucial to ensure appropriate management. It requires high-sensitivity, preferably live, cell-based assays (CBAs) for AQP4-IgG and MOG-IgG. Less sensitive or less specific methods, such as enzyme-linked immunosorbent assay (ELISA) or fixed CBAs, may yield false negative results, leading to misclassification of antibody-positive patients as double-seronegative and implications for prognosis and treatment. When clinical suspicion remains high despite negative results, repeating antibody testing after 3 to 6 months or during relapse is recommended, particularly if the initial test was performed during immunosuppression, remission, or after plasma exchange. 15 A slow rise in serum AQP4 antibody titers may occur before relapse, 16 underscoring the value of retesting with the most sensitive test available, preferentially CBA. In contrast, when testing is performed under optimal conditions (using live CBA, in the absence of immunosuppressive therapy, and during a relapse), a negative result is reliable, and repeat testing is generally unnecessary. 15

Long-term observational studies 2 3 indicate that relapses in NMOSD are most frequent within the first few years after disease onset, but late relapses are also well documented. Notably, discontinuation of therapy in AQP4-IgG-positive NMOSD has been consistently associated with high relapse rates and substantial risk of irreversible neurological damage. 3 4 17 Consequently, expert consensus 14 17 advocates for long-term or even lifelong immunosuppression in seropositive patients. For seronegative NMOSD, the absence of serological markers adds diagnostic and prognostic uncertainty, intensifying the challenge of deciding when—or whether—therapy withdrawal might ever be justified.

Although some patients fulfilling the NMOSD diagnostic criteria may experience long-term remission without recurrence, especially among seronegative individuals, these cases remain the exception. The rarity of sustained remission and the severity of potential relapses have led some experts to argue against treatment discontinuation. Nonetheless, these clinicians raise a legitimate clinical question: can selected patients with a clearly-monophasic course safely discontinue therapy after many years of stability? A few authors 18 19 20 have cautiously proposed that selected patients with extended remission (≥ 5–10 years), monophasic presentation, and no severe relapses or magnetic resonance imaging (MRI) progression could be considered for treatment withdrawal. The suggested criteria also include absence of LETM, lack of new symptoms, and favorable radiological evolution. 1 20 21 22 However, these proposals rest on anecdotal evidence or isolated retrospective case reports. Rare cases of prolonged spontaneous remission, some spanning nearly two decades, have been described, including in seropositive individuals. 18 19 Yet, these are exceptions and do not establish a safe paradigm. In most instances, relapse eventually occurs, often with devastating consequences. 1 2 3 4

Without serological markers, there are no validated tools to stratify relapse risk or guide cessation decisions. Biomarkers under investigation, such as glial fibrillary acidic protein (GFAP), neurofilament light chain (NfL), C-X-C motif chemokine 12 (CXCL12), and interleukin-6 (IL-6), may help fill this gap. 23 Elevated serum and cerebrospinal fluid (CSF) GFAP levels correlate with disease activity and may assist in relapse monitoring, but their predictive value still requires further investigation. 23 24 Although serum GFAP has repeatedly shown promise as a biomarker, the lack of standardized cutoff values and the predominance of data from AQP4-IgG–positive cohorts limit its clinical adoption. Therefore, these markers should be viewed as research tools rather than decision-making aids until robust validation occurs. Current guidelines 25 26 acknowledge the potential of serum GFAP, but they do not yet recommend its isolated use for therapeutic decision-making. A balanced interpretation is essential: emerging biomarkers help to illuminate NMOSD pathophysiology, but they should not prematurely drive treatment withdrawal.

Moreover, maintenance therapies such as rituximab, azathioprine, and mycophenolate are generally well tolerated. While they carry risks, the risk-benefit ratio still heavily favors continued use to prevent irreversible neurological injury. 2 13 Long-term immunosuppression inevitably carries cumulative burdens such as increased susceptibility to infection, possible oncologic risk, cytopenia, and reduced quality of life. These concerns underscore the importance of active monitoring and preventive care. Clinicians should employ proactive measures such as ensuring up-to-date vaccinations, screening for latent infections (such as tuberculosis and hepatitis), and monitoring for therapy-related malignancies. 27 Psychosocial aspects, such as chronic fatigue, medication adherence, and anxiety related to lifelong treatment, also need consideration and shared decision-making.

Experience from MS underscores the dangers of discontinuing therapy. The Discontinuation of Disease Modifying Therapies (DMTs) in Multiple Sclerosis (MS) (DISCOMS) trial, which followed stable older MS patients for 2 years after treatment discontinuation, demonstrated a significantly higher rate of relapse or new MRI lesions (of approximately 12.2%) compared to patients who continued treatment (4.7%). 28 Given NMOSD's propensity for severe, relapse-related disability, the risk of discontinuing maintenance immunotherapy in NMOSD is expected to be even greater. This strongly suggests that indefinite immunosuppressive treatment remains the safer approach in double-seronegative NMOSD, since even a single catastrophic relapse could outweigh the potential risks of long-term therapy.

Accordingly, treatment discontinuation should never be routine. In rare cases in which a patient expresses a desire to stop therapy shared decision-making is essential. The clinician's responsibility is to present the evidence and provide a transparent appraisal of risks. If withdrawal is pursued, it should only occur under close supervision, with scheduled neurological follow-up, MRI scans, and an emergency action plan in place. Rapid access to therapy reinitiation is critical in case of disease reactivation.

In conclusion, discontinuing immunosuppressive therapy in patients with NMOSD, including those who are double-seronegative and have achieved long-term remission, remains a high-risk strategy that should be approached with utmost caution. Accurate diagnosis through sensitive assays, recognition of disease heterogeneity, and balanced use of emerging biomarkers form the foundation of individualized care. There are currently no reliable clinical, imaging, or biological predictors that identify patients who can safely stop treatment. Until high-quality, prospective studies and validated biomarkers become available, the standard of care should continue to favor long-term maintenance therapy to minimize the risk of catastrophic relapse. In NMOSD, stability does not equal safety, and therapeutic continuity remains the surest defense against irreversible neurological damage.
